# Pitavastatin Differentially Modulates MicroRNA-Associated Cholesterol Transport Proteins in Macrophages

**DOI:** 10.1371/journal.pone.0159130

**Published:** 2016-07-14

**Authors:** Haijun Zhang, Brian D. Lamon, George Moran, Tao Sun, Antonio M. Gotto, David P. Hajjar

**Affiliations:** 1 Department of Cell and Developmental Biology, Weill Medical College of Cornell University, 1300 York Ave, New York, New York, 10065, United States of America; 2 Department of Genetic Medicine, Weill Medical College of Cornell University, 1300 York Ave, New York, New York, 10065, United States of America; 3 Department of Pathology and Laboratory Medicine, Weill Medical College of Cornell University, 1300 York Ave, New York, New York, 10065, United States of America; 4 Center of Vascular Biology, Weill Medical College of Cornell University, 1300 York Ave, New York, New York, 10065, United States of America; 5 Department of Medicine, Weill Medical College of Cornell University, 1300 York Ave, New York, New York, 10065, United States of America; The University of Tennessee Health Science Center, UNITED STATES

## Abstract

There is emerging evidence identifying microRNAs (miRNAs) as mediators of statin-induced cholesterol efflux, notably through the ATP-binding cassette transporter A1 (ABCA1) in macrophages. The objective of this study was to assess the impact of an HMG-CoA reductase inhibitor, pitavastatin, on macrophage miRNAs in the presence and absence of oxidized-LDL, a hallmark of a pro-atherogenic milieu. Treatment of human THP-1 cells with pitavastatin prevented the oxLDL-mediated suppression of miR-33a, -33b and -758 mRNA in these cells, an effect which was not uniquely attributable to induction of SREBP2. Induction of ABCA1 mRNA and protein by oxLDL was inhibited (30%) by pitavastatin, while oxLDL or pitavastatin alone significantly induced and repressed ABCA1 expression, respectively. These findings are consistent with previous reports in macrophages. miRNA profiling was also performed using a miRNA array. We identified specific miRNAs which were up-regulated (122) and down-regulated (107) in THP-1 cells treated with oxLDL plus pitavastatin versus oxLDL alone, indicating distinct regulatory networks in these cells. Moreover, several of the differentially expressed miRNAs identified are functionally associated with cholesterol trafficking (six miRNAs in cells treated with oxLDL versus oxLDL plus pitavastatin). Our findings indicate that pitavastatin can differentially modulate miRNA in the presence of oxLDL; and, our results provide evidence that the net effect on cholesterol homeostasis is mediated by a network of miRNAs.

## Introduction

MicroRNAs (miRNAs) are small, ~22 nucleotide sequences of RNA that regulate gene expression at the post-transcriptional level. miRNAs have been linked to cardiovascular disease via a plethora of pathophysiological events such as endothelial dysfunction, smooth muscle cell proliferation and migration, apoptosis, angiogenesis as well as alterations in cholesterol homeostasis-including HDL genesis, cholesterol efflux and reverse cholesterol transport [1]. While the individual influence of a miRNA on a single target may be modest, miRNAs function in concert, targeting multiple genes and/or steps which can mediate their regulatory effects. To this end, further studies into the identification of the network of miRNAs involved in the complex pathophysiological processes of atherogenesis are warranted.

The network of miRNAs linked to cholesterol homeostasis is extensive. In the liver, miR-22, -758, -185, -96, -223, -125a, and -144 modulate cholesterol efflux and homeostasis via the ATP-binding cassette transporter A1 (ABCA1), ABCB11, ATP8B1 and SR-B1 [2]. The first identified and most well characterized of these miRNAs is miR-33, which exists in two known forms that differ by two nucleotides (miR-33a and miR-33b). miR-33a and -33b have been demonstrated to physiologically impact the fine-tuning of cholesterol efflux in the liver, primarily through ABCA1 [3]. Furthermore, the genetic deletion of miR-33 in mice increases plasma HDL-C levels and reduces the progression of atherosclerosis [4]. While the bulk of evidence has focused on the role of miRNA in hepatocytes, recent evidence has shown that specific miRNAs are altered in macrophages, suggesting a possible role in arterial cell cholesterol homeostasis. ABCA1 influences the cholesterol efflux process in macrophages in the artery wall, and this reverse cholesterol transport in the body is critical to athero-protection by removing cholesterol from cells so that it can be transported back to the liver where it is re-used or excreted [5]. miR-33 and -758 have been specifically shown to negatively regulate ABCA1 in macrophages [2,4]. In early atherogenesis, macrophages are engulfed by modified LDLs such as oxidized LDL (oxLDL) which results in the formation of atherogenic “foam” cells. Importantly, oxLDL promotes several other pro-atherogenic processes including monocyte attraction, vasoconstriction, cell proliferation and plaque instability [6]. Less is known about the impact of oxLDL on macrophage miRNA expression and how these suppressors differentially exert their target effects in this type of atherogenic environment.

Recent evidence also suggests that miRNAs may play a role in mediating the beneficial pleiotropic effects observed with statin-based lipid-lowering therapies [7]. Pitavastatin competitively inhibits HMG-CoA reductase; and, like others in its class, it has been linked to a variety of athero-protective effects independent of this primary mechanism of action [8]. For example, our group has previously reported that pitavastatin increases SRB1, HDL binding, and efflux of labeled cholesterol to HDL in macrophages [9]. Pitavastatin has also been shown to regulate VCAM-1 through miR-126 in endothelial cells [10]; and, others have demonstrated that several statins increase miR33 expression, and decrease ABCA1 expression and cholesterol efflux in macrophage cell lines [10,11]. Thus, the rationale for miRNAs mediating the cholesterol regulatory effect of statins is reasonably robust. The present study was designed to test the hypothesis that a specific statin, pitavastatin, can modulate a network of miRNAs in macrophages, and that the influence of this statin on these regulatory networks is altered in a lipid environment designed to mimic early atherogenesis.

## Materials and Methods

### Cell culture and treatment protocols

Cells of the THP-1 human monocytic cell line were purchased from ATCC (Rockville, MD) and cultured in RPMI 1640 complete medium containing 10% fetal calf serum, penicillin and streptomycin. Cells were adjusted to a density of 2.5x10^6^/cm2 and transferred to 60 x 15mm dishes. PMA (Sigma) was added to the complete media (200nM) to induce monocyte differentiation to macrophages. After 24 hours, the PMA was removed and replaced with complete media. After 3–5 hours, four dishes of cells received four separate treatments: 1) control, RPMI complete media; 2) 50ug/mL oxidized LDL purchased from Intracel (Frederick Maryland) in RPMI complete media; 3) 10 uM pitavastatin from Kowa Pharmaceuticals (Nagoya, Japan) in RPMI complete media; 4) 50ug/mL oxidized LDL and 10 uM pitavastatin in RPMI complete media. All groups were treated side-by-side for 8, 16 and 24 hours. We used this amount of oxLDL and pitavastatin for 16hrs since this proved to be optimal in pilot studies.

### Isolation of Total Cellular RNA and Real Time RT-PCR (qRT-PCR)

After treatment, cells in each treatment group were lysed using TRIzol reagent (Invitrogen, Carlsbad, CA), including a 5-minute incubation in the TRIzol. Lysates were then mixed well with chloroform and incubated for 3 minutes, after which the mixtures were centrifuged for 15 minutes at 12,000 x g at 4°C. The aqueous phase lysates were removed and mixed with isopropanol to precipitate total cellular RNA. ABCA1, SREBP2 and miRNA mRNA expression was measured by real time qRT-PCR, using SYBR green PCR master mix (Applied Biosystems) with the following primer sets: hsa-ABCA1-qPCR-F: caacagtttgtggccctttt, hsa-ABCA1-qPCR-R: gacaaacacagctggcaaga; hsa-SREBP2-qPCR-F: atcgctcctccatcaatgac, hsa-SREBP2-qPCR-R: ttcctcagaacgccagactt; hsa-mir-33a-qPCR-F: agttgcattgcatgttctgg, hsa-mir-33a-qPCR-R: ccttcagtcagggcagtctc; hsa-mir-33b-qPCR-F: ggtgcattgctgttgcatt, hsa-mir-33b-qPCR-R: ctctgggaggggcaggat; hsa-mir-758-qPCR-F: gaccagagagcacacgcttt, hsa-mir-758-qPCR-R: tggccccagtttactgtctc; has-GAPDH-qPCR-F caatgaccccttcattgacc, hsa-GAPDH-qPCR-R gacaagcttcccgttctcag. GAPDH mRNA was used to normalize mRNA expression.

### Protein and Western Blot analysis

After treatment, cells were washed once with PBS and lysed with cold lysis buffer (50nM Tris, pH 7.5; 150nM NaCl, 1% Triton-X 100, 1% sodium deoxycholate, 1mM PMSF, and 10 ug/mL Calbiochem (La Jolla, CA) Protease Inhibitor Cocktail Set III). After 45 minutes, lysates were sonicated on ice for 3 cycles of 3 seconds each; and centrifuged at 4°C for 15 minutes at 16.1x103 x g. Supernatants were saved at -80C until the concentration of whole protein extract was determined. For whole cell lysates, cells were washed with PBS and scraped into ice-cold lysis buffer (50 mM Tris-HCl, pH 7.5, 150 mM NaCl, 1% Triton X-100, 1 mM sodium fluoride, 1 mM sodium orthovanadate, and complete protease inhibitor cocktail (Sigma)). Cells were rotated on an orbital shaker at 4°C for 15 minutes and then spun at 13,000 RPM. The supernatant was collected immediately and protein concentration was determined using the Lowry method [12].

Protein lysates from each of the four treatment groups were loaded into 7.5% (for ABCA1) and 10% (for SRB1 and SREBP2) acrylamide SDS-PAGE gels and run via electrophoresis. The proteins were transferred to PVDF Transfer Membrane from Thermo Scientific (Rockford, IL) overnight at 4°C and then blocked in a solution containing 0.1% Tween 20/PBS (PBS-T) and 5% dry nonfat milk for 1 hour. Membranes were then incubated overnight with the corresponding antibodies: rabbit polyclonal from Novus Biologicals, Littleton, CO for ABCA1, SRB1, and SREBP2, and goat polyclonal from Santa Cruz Biotechnology, Dallas, TX for the actin control, 1:1000 in 2.5% milk in PBS-T. Membranes were subsequently washed in PBS-T and incubated with 1:3000 HRP conjugates containing goat anti-rabbit (Bio-Rad, Hercules, CA for ABCA1, SRB1, and SREBP2; conjugates containing donkey anti-goat (Santa Cruz) for Actin) in 1% milk in PBS-T for one hour. After a final wash, membranes were incubated in chemiluminescence reagents (Thermo Pierce ECL or General Electric Amersham ECL) and then developed.

### miRNA microarray

TRIzol extracted RNAs were quantified by a spectrometer and assessed for sample quality by checking the RNA integrity through an agarose gel electrophoresis. miRNA microarray analyses were performed using Human miRNA Array (Version miRHuman_20, LC Sciences, Houston, TX), which included 2555 unique probes designed for mature miRNAs based on Sanger miRBase Release 20. The image files from microarrays were processed and the signals were background subtracted and normalized according to the standard protocol of LC Sciences. T-test was used to identify the differentially expressed miRNAs among groups. Transcripts between groups (p-value < 0.01) were defined as differentially expressed transcripts. Mature miRNAs were sorted separately according to differential ratios. The ratio values were presented in log2 scale for easy assessing differential direction as well as magnitude. A positive log2 value indicates up-regulation and a negative log2 value indicates down-regulation.

### Statistical analysis

Data are presented as the mean ± standard error (S.E.M.) and analyzed by ANOVA followed by the Newman-Keuls post hoc test. The null hypothesis was rejected at P<0.05. Real Time PCR data is expressed as arbitrary units and mRNA normalized to GAPDH. Densitometric analysis of western blotting is expressed as protein of interest to actin ratio or fold increase over control after correction for equal loading by actin.

## Results

### Regulation of miR-33 and -758 by pitavastatin in the presence and absence of oxLDL

**Fig 1:**
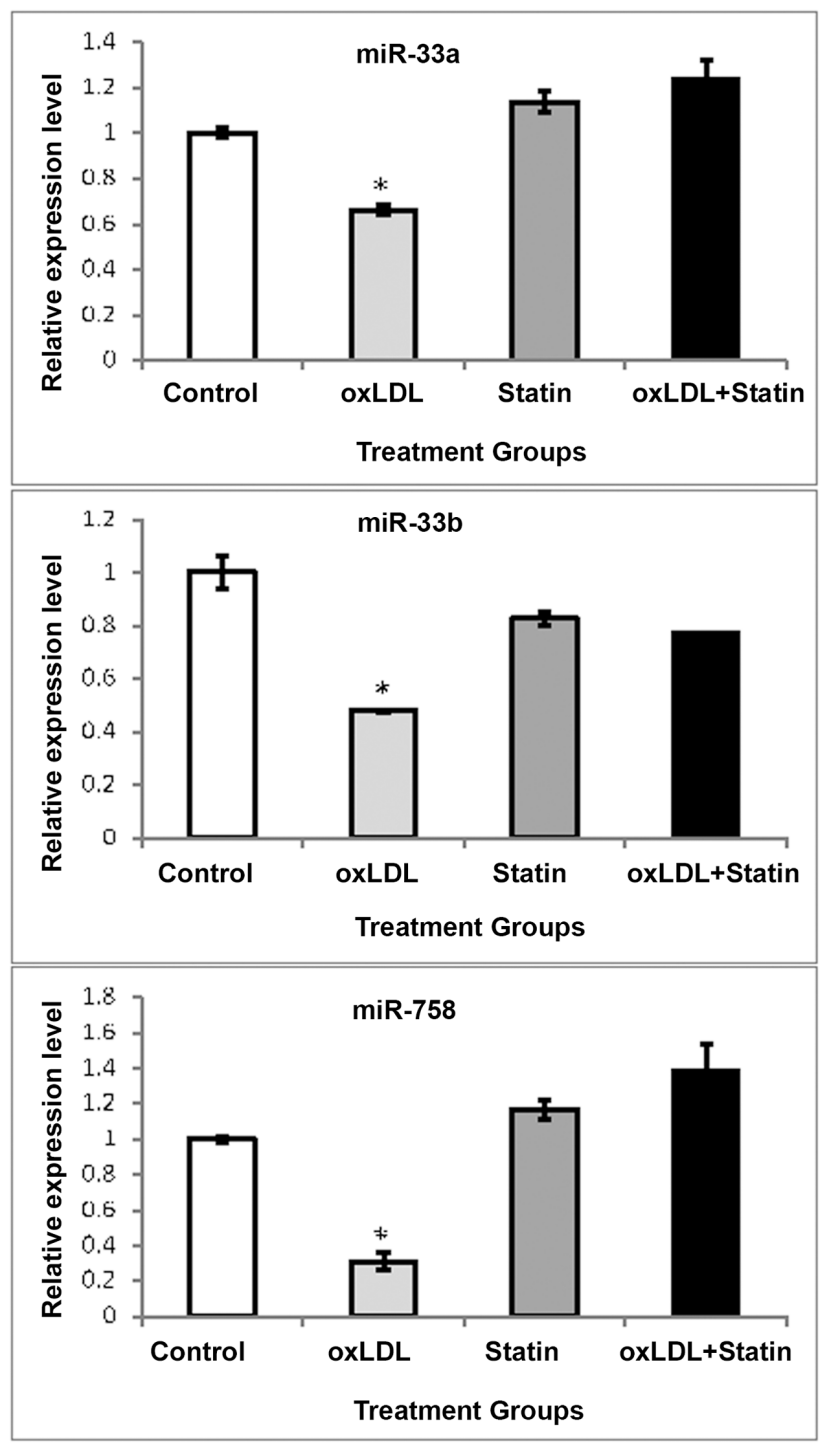
Expression of microRNAs in Macrophages. Expression of miR-33a, -33b and -758 in THP-1 macrophages were measured by real time RT-PCR. Each miRNA was assessed in the presence of oxidized LDL (oxLDL) (50ug/mL), pitavastatin (Statin) (10uM), or the combination. GAPDH mRNA was used to normalize mRNA expression and data are expressed as arbitrary units. oxLDL and pitavastatin alone groups were compared to control (*P<0.05) while the oxLDL+pitavastatin group was compared to oxLDL (**P<0.05).

A human monocyte cell line (THP-1) was selected to first investigate the impact of oxLDL on the pitavastatin-mediated regulation of miRNAs, which have been previously linked to key cholesterol homeostasis and efflux proteins. As shown in Fig 1, oxLDL elicited a significant reduction in both miR-33a and -33b mRNA after 16 hours treatment. Pitavastatin alone led to a modest increase in miR-33a and a modest decrease in miR-33b in the control group, neither of which reached statistical significance. However, pitavastatin prevented the decreases in miR-33a and in miRNA33b seen in the oxLDL-alone group. In a pattern similar to miR-33, miR-758 expression was suppressed by oxLDL. While pitavastatin alone did not significantly alter miR-758, this drug prevented the suppressive effects of oxLDL on miR-758 in these macrophages.

### Regulation of SREBP-2 by pitavastatin in the presence and absence of oxLDL

**Fig 2:**
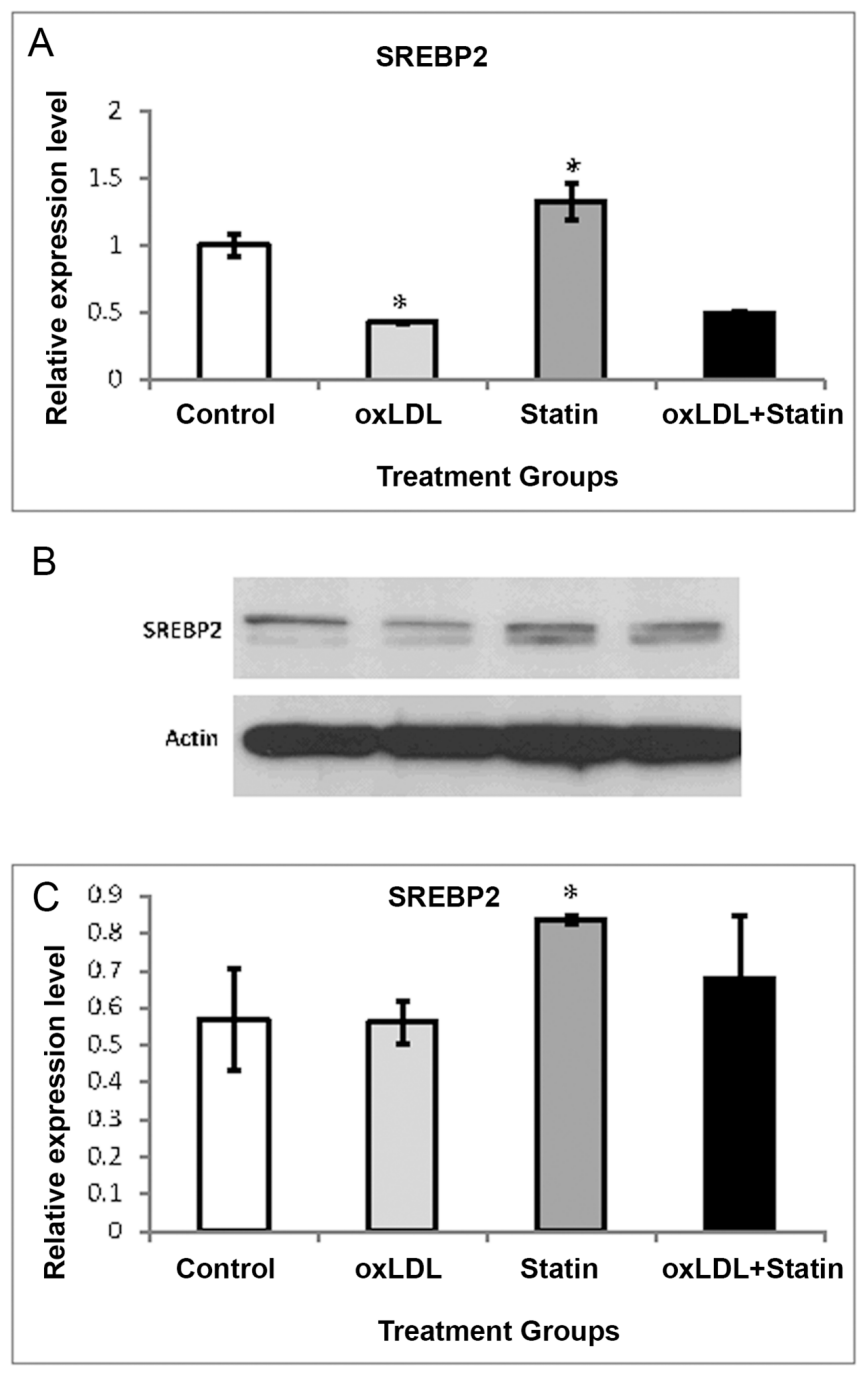
Expression of SREBP-2 in Macrophages. (A) This regulator of cholesterol metabolic genes and known to harbor miR-33a and -33b, was examined in THP-1 macrophages by real time RT-PCR. SREBP-2 was assessed in the presence of oxidized LDL (oxLDL) (50ug/mL), pitavastatin (Statin) (10uM), or a combination of the two. GAPDH mRNA was used to normalize mRNA expression, and data are expressed as arbitrary units. (B and C) SREBP-2 protein expression was assessed and quantified using densitometric analysis by normalization with actin. In all cases, oxLDL and pitavastatin alone groups were compared to the control group (*P<0.05) while the oxLDL+pitavastatin group was compared to the oxLDL group (**P<0.05).

Sterol regulatory element binding protein (SREBP) is a lipogenic transcription factor; and, miR-33a and miR-33b are intronic miRNAs within SREBP. SREBP-2 is known to be induced by various statins [13], and consistent with previous findings, we observed an increase in SREBP-2 mRNA by pitavastatin (Fig 2). Conversely, oxLDL suppressed SREBP-2 mRNA and a combination of pitavastatin and oxLDL nullified the stimulatory effect of pitavastatin on SREBP-2 expression. Protein expression of SREBP-2 was not suppressed in the presence of oxLDL despite the observed effect on mRNA; however, the suppressive effect was partially inhibited by pitavastatin.

### Time points of oxLDL treatment on cells

Because expression levels of miR-33a and miR-33b were altered significantly after 16 hours treatment of oxLDL, we questioned whether miR-33 expression also is changed at other time points. THP-1 cells were treated with oxLDL for 8, 16 and 24 hours. Expression of miR-33a, miR-33b and miR-758 displayed significant reduction from 8 to 16 hours (S1 Fig). Moreover, SREBP expression had a slight change from 8 to 16 hours, and a reduction from 16 to 24 hours (S1 Fig). These results suggest that treatment at 16 hours is an optimal time point to evaluate miRNA expression.

### Regulation of ABCA1 by pitavastatin in the presence and absence of oxLDL

**Fig 3:**
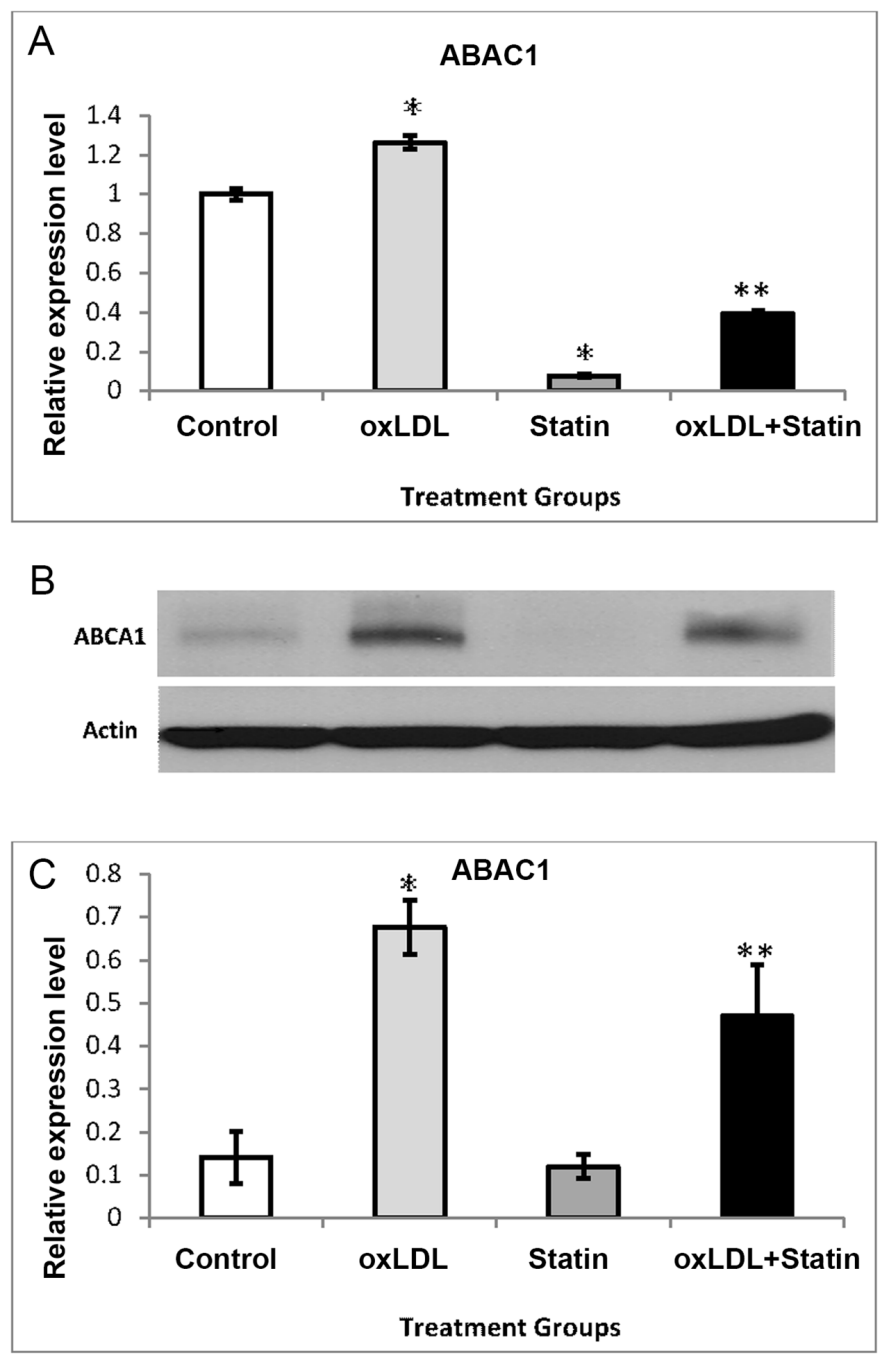
Expression of ABCA1 in Macrophages. (A) ATP-binding cassette transporter ABCA1 is a major cholesterol efflux regulatory protein previously demonstrated to be regulated by miRNAs. mRNA expression of ABCA1 in THP-1 macrophages was assessed in the presence of oxidized LDL (oxLDL) (50ug/mL), Pitavastatin (Statin) (10uM), or a combination of both. GAPDH mRNA was used to normalize mRNA expression; and, the data are expressed as arbitrary units. (B and C) ABCA1 protein expression was assessed and quantified using densitometric analysis with normalization by actin. In all cases, oxLDL and pitavastatin groups were compared to control groups (*P<0.05) while the oxLDL+pitavastatin group was compared to the oxLDL group (**P<0.05).

miR-33a and miR-33b were the first reported miRNAs to be linked to ABCA1 [2]. Ergo, we examined the effects of oxLDL, pitavastatin, and pitavastatin + oxLDL on ABCA1 mRNA levels in human THP-1 cells. Shown in Fig 3, oxLDL increased ABCA1 mRNA while pitavastatin alone suppressed ABCA1 mRNA expression by more than 90%. The effect of oxLDL (increase) and pitavastatin (decrease) in ABCA1 mRNA is consistent with previous reports in other macrophages cell lines and with other statins [13–16]. In the presence of oxLDL, pitavastatin decreased ABCA1, but not to the extent observed with pitavastatin alone. Consistent with our mRNA data, expression of ABCA1 protein was induced by oxLDL. Interestingly, the suppressive effect of pitavastatin on ABCA1 mRNA was not reflected in the protein expression analysis although baseline expression was modest. However, pitavastatin did cause a 30% reduction in oxLDL-mediated induction of ABCA1 (Fig 3), indicative of competing metabolic influences on ABCA1.

### Expression of miRNAs in THP-1 cells challenged with oxLDL and pitavastatin

Having shown that miRNAs with an established link to cholesterol homeostasis (miR-33 and miR-758) are differentially targeted by pitavastatin in the presence and absence of oxLDL, we set out to define if a broader network of miRNAs is similarly modulated. To identify miRNAs responding to oxLDL or pitavastatin, we preformed miRNA profiling using THP-1 cells that were either: untreated (control), treated with oxLDL only, treated with pitavastatin only, or treated with oxLDL plus pitavastatin. We selected miRNAs with differential expressions in our cells (*p* < 0.01) in different experimental groups. Compared to controls, 123 miRNAs were up-regulated and 71 were down-regulated in THP-1 cells treated with oxLDL; 112 were up-regulated and 47 were down-regulated in cells treated with pitavastatin; 111 were up-regulated and 95 were down-regulated in cells treated with oxLDL plus pitavastatin. Moreover, 26 miRNAs were up-regulated and 34 were down-regulated in THP-1 cells treated with pitavastatin versus oxLDL; 122 were up-regulated and 107 were down-regulated in cells treated with oxLDL plus pitavastatin versus oxLDL; and of high interest, 112 were up-regulated and 112 were down-regulated in cells treated with oxLDL plus pitavastatin versus pitavastatin alone. Furthermore, we analyzed genome-wide miRNA expression patterns in control THP-1 cells, and cells treated with oxLDL, pitatvastatin or oxLDL plus pitavastatin using the unsupervised hierarchical clustering technique. Distinct expression patterns of miRNAs were observed among these four groups, suggesting significant miRNA profiling changes upon cellular exposure to oxLDL, pitavastatin or the combination.

Among differentially expressed miRNAs, we found nine miRNAs that are functionally associated with cholesterol trafficking, and one miRNA (miR-16-2-3p) that is THP-1 cell-specific (Table 1). Moreover, we detected seven miRNAs that displayed the most significantly differential expression among samples which had the highest signals in the microarray, including miR-33b-3p in this study (Table 1). Among these miRNAs, eight were differentially expressed between control THP-1 cells and cells treated with oxLDL, four between the control cells and cells treated with pitavastatin, and five between the control cells and cells treated with oxLDL plus pitavastatin. Furthermore, two miRNAs were differentially expressed between cells treated with oxLDL versus pitavastatin, six between cells treated with oxLDL versus oxLDL plus pitavastatin, and four between cells treated with pitavastatin versus oxLDL plus pitavastatin (Table 1).

**Table 1:** Functions of miRNAs displaying significantly differential expression.

miRNA	Samples Compared	*p* value	Change	Functions of miRNA
**Cholesterol trafficking-related**				
miR-126-3p	Control, oxLDL	3.65E-03	Down	Reduced circulating levels seen in patients with coronary artery disease; reduced expression in atherosclerotic lesions; promotes CXCL12 expression; inhibit leukocyte
	Control, Pitavastatin	6.47E-03	Down	
	Control, oxLDL+pitavastatin	1.32E-03	Down	
miR-378a-3p	Control, oxLDL	1.49E-03	Down	Increases the transcriptional activity of CCAAT-enhancer-binding proteins (or C/EBPs) α and β on adipocyte gene promoters; increase in response to adipocyte differentiation
miR-221-5p	Control, oxLDL	4.53E-03	Up	Contributes to SMC dedifferentiation
	oxLDL, oxLDL+pitavastatin	1.47E-04	Down	
	Pitavastatin, oxLDL+pitavastatin	5.07E-04	Down	
miR-21-5p	Control, oxLDL+pitavastatin	4.81E-04	Up	Essential for PDGF-mediated cell proliferation, by repressing c-Kit
miR-23a-3p	Control, oxLDL	9.37E-03	Up	Essential for pathological angiogenesis, by promoting angiogenic signaling through targeting Sprouty2 ans the transmembrane semaphorin protein Sema6A
	oxLDL, oxLDL+pitavastatin	8.56E-03	Down	
miR-23b-3p	Control, oxLDL	8.28E-03	Up	
	oxLDL, oxLDL+pitavastatin	8.80E-03	Down	
miR-23c	oxLDL, oxLDL+pitavastatin	8.44E-03	Down	
miR-27a-3p	Control, oxLDL	8.92E-03	Down	
miR-27b-3p	Control, oxLDL	3.47E-03	Up	
	Control, Pitavastatin	1.06E-03	Down	
	Control, oxLDL+pitavastatin	2.40E-03	Up	
	oxLDL, Pitavastatin	2.76E-04	Down	
	Pitavastatin, oxLDL+pitavastatin	2.46E-04	Up	
**THP-1 specific**				
miR-16-2-3p	Control, oxLDL+pitavastatin	8.94E-04	Down	Targets cyclooxygenase-2; correlated with PBMCs
	oxLDL, oxLDL+pitavastatin	5.02E-04	Down	
	Pitavastatin, oxLDL+pitavastatin	1.28E-03	Down	
**Most significantly different and highest signal in microarray**				
miR-18b-5p	Control, oxLDL	6.89E-05	Down	Implicated in variety of cancers (part of miR-17 microRNA precursor family)
miR-4326	Control, Pitavastatin	7.41E-05	Up	
miR-106a-5p	Control, oxLDL+pitavastatin	2.14E-04	Down	Implicated in variety of cancers (part of miR-17 microRNA precursor family)
miR-374a-5p	oxLDL, Pitavastatin	3.80E-05	Down	
miR-221-5p	oxLDL, oxLDL+pitavastatin	1.47E-04	Down	Targets CD117, which prevents cell migration and proliferation in endothelial cells; known as an anti-angiogenic miRNA: involved in induction of angiogenesis, expressed in liver cancer, inducing tumor angiogenesis
miR-664b-3p	Pitavastatin, oxLDL+pitavastatin	1.17E-04	Down	
miR-33b-3p	Control, Pitavastatin	3.53E-03	Up	Represses ABCA1 expression to regulate cholesterol efflux to apolipoprotein A1

Functions of miRNAs displaying significantly differential expression and high expression levels detected by microarrays. miRNAs that are functionally associated with cholesterol trafficking in THP-1 cells are included in Table 1 which showed the most significantly differential expressions among treatment groups. Treatment groups include control, oxidized LDL (50ug/mL), pitavastatin (10uM) or the combination of the latter.

Moreover, we searched potential targets for miR-33 and miR-758 using the Targetscan (http://www.targetscan.org/) according to their seed sequences, which have significant silencing effects on target genes (S1 Table). The functions of target genes were further searched in GeneCards (http://www.genecards.org/) (S1 Table). Many of the target genes are involved in cholesterol transport, which is consistent with our observations (Fig 1).

## Discussion

We have previously reported that pitavastatin supports anti-atherogenic mechanisms such as suppression of oxLDL uptake and foam cell formation by targeting the oxLDL receptor CD36 [14], enhancement of anti-inflammatory actions in macrophages [15], and changes in arterial homeostasis by favoring fibrinolysis over thrombosis [16]. There is emerging evidence that networks of miRNAs may function as critical intermediaries, or at a minimum, modulators of the pleiotropic effects of statins during atherogenesis. In the present study, we provide evidence that pitavastatin differentially modifies miRNAs associated with the cholesterol-trafficking protein ABCA1 in the presence of oxidized LDL.

Under baseline conditions, we observed that oxLDL decreased miR-33a, miR33b, and miR-758. Importantly, pitavastatin prevented the suppression of miR-33a, miR33b, and miR-758 by oxLDL (Fig 1). To our knowledge, this is the first report to demonstrate the differential modulation of miRNAs in presence of oxLDL by a statin. Previous reports have shown that atorvastatin and simvastatin induce miR33a mRNA in unloaded cells, and to a lesser degree, in cholesterol-loaded cells [17]. The increase in miR-33 has been linked to induction of SREBP-2 [18]. In humans, miR-33a and -33b are encoded in the introns of SREBF-2 and SREBF-1, the former of which encodes for SREBP-2, and is critical to cholesterol homeostasis. Although we observed a clear induction of SREBP mRNA and protein by pitavastatin (Fig 2), they were not accompanied by an equally notable increase in miR-33. Therefore, the elevated levels of miR-33 may not be necessarily attributed to SREBP-2. At this time, the mechanism of these findings remains unclear. In the presence of oxLDL, pitavastatin was unable to elicit the stimulatory effect on SREBP mRNA or protein. Interestingly, protein expression of SREBP-2 was not suppressed in the presence of oxLDL notwithstanding the observed effect on mRNA. Such findings indicate that the effect could be on RNA translation or degradation.

The impact of statins in regulating miR-33 and -758 under basal conditions appear to be modest, however this pathway becomes amplified in a pro-atherogenic milieu when oxLDL is present. It appears consistent that these two miRNAs respond similarly as pitavastatin prevented the suppressive effects of oxLDL. These miRNAs have been linked to a decrease in ABCA1; therefore, it is plausible that the statins may generate an inhibitory effect on ABCA1 by preventing the reduction of a negative regulator of its expression in a pro-atherosclerotic milieu. Based on our findings and the existing literature, it is unlikely that miR-33 is solely responsible for regulating of ABCA1 and other RCT proteins, as the slight increase in miR-33 cannot rationally explain the robust suppressive effect on ABCA1 by pitavastatin (Fig 3). The inhibitory effect of statins on ABCA1 has been described with various statins to be dose- and time-dependent, thus our findings can be extrapolated to the findings of others [19]. Collectively, these findings confirm the positive (oxLDL) and negative (pitavastatin) influence that these effectors have on the regulation of ABCA1 in THP-1 cells [13,19,20]

As the oxLDL-mediated induction of ABCA1 was blunted by pitavastatin (Fig 3), it is interesting to note that small increase in ABCA1 mRNA (~20%) with oxLDL resulted in a much more robust (over 4- fold) increase in protein. Similarly, the ability of pitavastatin to suppress ABCA1 when oxLDL was present was more modest compared to the statin alone. Of note, the effect of oxLDL is distinct from cholesterol which was demonstrated to reverse the statin-mediated down-regulation of ABCA1 [13]. Nevertheless, our findings support the notion that ABCA1 expression is linked to oxLDL and provide evidence that the association may be linked to alterations in miRNA expression [19].

The final objective of these studies was to begin to understand our findings and related reports in the literature in the context of the complexity of regulatory networks of miRNAs. To accomplish this objective, we implemented a hypothesis-generating approach using the human miRNA Array platform. The overarching potential of cardiovascular-related events associated with the differentially expressed miRNAs in THP-1 macrophages are apparent. For example, has-miR-126-3p has been shown to be associated with the coronary heart disease and atherosclerotic lesions, has-miR-221-5p has been associated with SMC de-differentiation, and has-miR-23a-3p, has-miR-23b-3p, has-miR-23c, has-miR-27a-3p, and has-miR-27b-3p have been shown to be involved with the progression of angiogenesis (Table 1).

miRNA microarray analysis has identified differentially expressed miRNAs that are involved in multiple functions in cholesterol metabolism (e.g. 6 cholesterol trafficking-related miRNAs in cells treated with oxLDL versus oxLDL plus pitavastatin, and thus highly relevant to cardiovascular disease. Specific miRNAs were up-regulated (122) and down-regulated (107) in THP-1 cells treated with oxLDL plus pitavastatin versus oxLDL alone, indicative of distinct regulatory networks in these cells. Moreover, several of the differentially expressed miRNAs identified are functionally associated with cholesterol trafficking (six miRNAs in cells treated with oxLDL versus oxLDL plus pitavastatin). These findings support the notion that the altered lipid environment in early atherogenesis, modeled by oxLDL, is critical to understanding the influence of statins on regulatory networks of miRNAs and cholesterol transport. Moreover, statin effects on cholesterol trafficking are tissue specific. Often, they will decrease ABCA1 in arterial macrophages, but in liver, they can increase its expression [20]. Niesor et al. have emphasized that if the statin effects on plasma levels of HDL are predominantly influenced by their effects on ABCA1 expression, this could explain the clinical findings that statin treatment slightly increases plasma HDL, notwithstanding our observations that pitavastatin can reduce macrophage ABCA1 [14]. Mao et al. [21] recently demonstrated that miR-33 expression can be upregulated in human THP-1 monocytes by inflammation, leading to a decrease in ABCA1 expression and cholesterol efflux. Moreover, we found an increase in SREBP-2 mRNA by pitavastatin (Fig 2), suppression of SREBP-2 by oxLDL. Further research is needed to define the cellular interactions between the statins and SREBPs.

Statins have been heralded for several therapeutic effects involving a vast number of molecular targets which complement their ability to inhibit HMG-CoA reductase and reduce serum cholesterol [7]. Our new findings presented herein provide evidence that pitavastatin can alter the capacity of oxLDL to suppress miR-33a, -33b, and -758 miRNAs in THP-1 cells. While the present study focused on the effect of pitavastatin, the effects of other statins on miR-33 and ABCA1 have been shown consistent enough to postulate a similar response profile across the class [3,17]. The rationale for our study is built in part on the assumption that a patient population receiving statins would be expected to have pro-atherogenic milieu in portions of the vascular wall, thus oxLDL mimics early phase atherosclerosis development. Our studies were aimed to help elucidate these pathways; and, our data support the concept that modulation of miRNAs by statins can impact the potential benefit of HDL-raising agents in patients receiving statins as recently reported [17].

## Supporting Information

S1 Fig Expression of miR-33a, -33b, -758 and SREBP-2 in THP-1 macrophages. (DOCX)

S1 Table Predicted targets for miR-33 and miR-758. (DOCX)
