# Supplementary material for: Pitavastatin Differentially Modulates MicroRNA-Associated Cholesterol Transport Proteins in Macrophages
Source: PLoS One. 2016 Jul 14;11(7):e0159130. doi: 10.1371/journal.pone.0159130 (PMC4945056; doi:10.1371/journal.pone.0159130)

**Pitavastatin Differentially Modulates MicroRNA-Associated Cholesterol Transport Proteins in Macrophages**

Haijun Zhang^1,2,#^, Brian D. Lamon^3,4,#^, George Moran^3,4^, Tao Sun^1^, Antonio M. Gotto Jr.^5^, David P. Hajjar^3,4,*^

1 Department of Cell and Developmental Biology, 2 Department of Genetic Medicin, 3 Department of Pathology and Laboratory Medicine, 4 Center of Vascular Biology, 5 Department of Medicine, Weill Medical College of Cornell University, 1300 York Ave, New York, NY 10065, USA.

*** Corresponding Author:** David P. Hajjar, Department of Pathology and Laboratory Medicine, Center of Vascular Biology, Weill Cornell Medical College, 1300 York Ave, New York, NY 10065

E-mail [dphajjar@med.cornell.edu](mailto:dphajjar@med.cornell.edu) ; tel: 212-746-6720; fax: 212-746-8789

# These authors contributed equally to the work in this study as first authors.

**Grants and Support:** This work was supported by a sponsored research agreement with KOWA Co, Ltd, Tokyo, Japan, and in part, by an R01-MH083680-08 grant from the NIH/NIMH (T.S.).

**Conflict of Interest Statement:** Drs. David P. Hajjar and Antonio M. Gotto Jr. were consultants for KOWA Pharmaceuticals in the area of atherosclerosis research.

**Figure legend**

Fig S1. Expression of miR-33a, -33b, -758 and SREBP-2 in THP-1 macrophages were measured by real time RT-PCR after treatment of oxidized LDL (50ug/mL) at 8, 16 and 24 hours. The relative values were normalized with their expression in control cells at the same time point.

Fig S1.


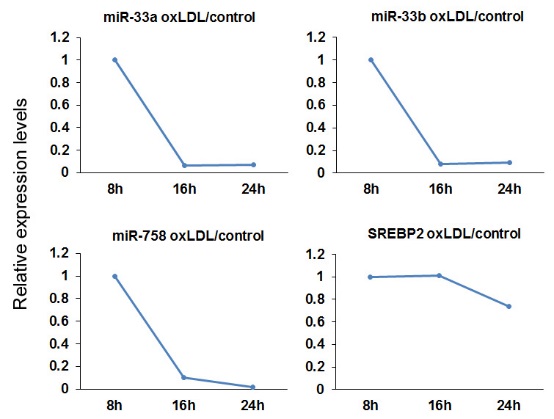

Supplement: S1 Fig — (DOCX) [file pone.0159130.s001.docx]
