# Supplementary material for: Pitavastatin Differentially Modulates MicroRNA-Associated Cholesterol Transport Proteins in Macrophages
Source: PLoS One. 2016 Jul 14;11(7):e0159130. doi: 10.1371/journal.pone.0159130 (PMC4945056; doi:10.1371/journal.pone.0159130)
Supplement: S1 Table — (DOCX) [file pone.0159130.s002.docx]

**S1 Table. Predicted targets for miR-33 and miR-758.**

| **Target symbol** | **Target gene name** | **Function of target gene** |
| --- | --- | --- |
| miR-33 | | |
| LIPI | Lipase, member I | The protein encoded by this gene is a phospholipase that hydrolyzes phosphatidic acid to produce lysophosphatidic acid. Defects in this gene are a cause of susceptibility to familial hypertriglyceridemia. |
| NPC1 | Niemann-Pick disease, type C1 | Mediates intracellular cholesterol trafficking via binding of cholesterol to its N-terminal domain |
| ABCA1 | ATP-binding cassette, sub-family A (ABC1), member 1 | Cholesterol efflux pump in the cellular lipid removal pathway. Mutations in this gene have been associated with Tangier's disease and familial high-density lipoprotein deficiency. |
| NCEH1 | Neutral cholesterol ester hydrolase 1 | May be responsible for cholesterol ester hydrolysis in macrophages, thereby contributing to the development of atherosclerosis |
| MSR1 | Macrophage scavenger receptor 1 | Mediate the endocytosis of modified low density lipoproteins (LDLs). |
| AGO2 | Argonaute RISC catalytic component 2 | Binding of RISC to a perfectly complementary mRNA generally results in silencing due to endonucleolytic cleavage of the mRNA specifically by AGO2. |
| ABCG5 | ATP-binding cassette, sub-family G (WHITE), member 5 | Appears to play an indispensable role in the selective transport of the dietary cholesterol in and out of the enterocytes and in the selective sterol excretion by the liver into bile. |
| SREBF1 | Sterol regulatory element binding transcription factor 1 | Regulates transcription of the LDL receptor gene as well as the fatty acid and to a lesser degree the cholesterol synthesis pathway. |
| SCAP | SREBF chaperone | Escort protein required for cholesterol as well as lipid homeostasis. |
| PRKAA1 | Protein kinase, AMP-activated, alpha 1 catalytic subunit | Regulates fatty acid and cholesterol synthesis. |
| miR-758 | | |
| ABCA1 | ATP-binding cassette, sub-family A (ABC1), member 1 | Cholesterol efflux pump in the cellular lipid removal pathway. Mutations in this gene have been associated with Tangier's disease and familial high-density lipoprotein deficiency. |
| ABCB11 | ATP-binding cassette, sub-family B (MDR/TAP), member 11 | Mutations in this gene cause a form of progressive familial intrahepatic cholestases which are a group of inherited disorders with severe liver disease from early infancy. |

Potential targets for miR-33 and miR-758 were searched using the Targetscan (http://www.targetscan.org/) according to their seed sequences. The functions of target genes were further searched in GeneCards (http://www.genecards.org/).
